# *Hc*TTR: a novel antagonist against goat interleukin 4 derived from the excretory and secretory products of *Haemonchus contortus*

**DOI:** 10.1186/s13567-019-0661-z

**Published:** 2019-06-04

**Authors:** XiaoWei Tian, MingMin Lu, WenJuan Wang, CaiWen Jia, Ehsan Muhammad, RuoFeng Yan, LiXin Xu, XiaoKai Song, XiangRui Li

**Affiliations:** MOE Joint International Research Laboratory of Animal Health and Food Safety, College of Veterinary Medicine, Nanjing Agricultural University, Nanjing, 210095 China

## Abstract

*Haemonchus contortus* (*H. contortus*) has evolved sophisticated evasion mechanisms to ensure their survival, including generating excretion and secretion products (ESPs) to regulate the secretion of host cytokines. Interleukin 4 (IL4) is a classic T-helper cell type 2 (Th2)-type cytokine that plays an irreplaceable role against nematode infection. In this study, three proteins, glutathione *S*-transferase domain containing protein (*Hc*GST), transthyretin domain containing protein (*Hc*TTR) and calponin actin-binding domain containing protein (*Hc*Cab), were identified to bind to goat IL4 by co-immunoprecipitation (Co-IP) assays and yeast two-hybrid screening. Additionally, cell proliferation analysis showed that *Hc*TTR blocked the IL4-induced proliferation of peripheral blood mononuclear cells in goats, while *Hc*GST and *Hc*Cab did not. In addition, *Hc*TTR could also downregulate the transcription of candidate genes in the IL4-induced JAK/STAT pathway. These results indicated that *Hc*TTR is a novel antagonist against goat IL4 from *Hc*ESPs, and this information could improve our understanding of the relationship between host cytokines and parasite infections.

**Electronic supplementary material:**

The online version of this article (10.1186/s13567-019-0661-z) contains supplementary material, which is available to authorized users.

## Introduction

Parasitic nematodes have evolved complex mechanisms to participate in host immunomodulatory and to evade host immune surveillance. During infection, these organisms have not simply warded off the host immune attack; but rather, nematodes interfere with, direct and modulate immune responses in favor of their own survival. Actually, nematodes have evolved immunosuppressive or immunomodulatory molecules to influence the function of cytokines, which can play an important role in protecting the host from infection of pathogens including parasites [1].

Recent studies have revealed potential relationships between host cytokines and parasite ESPs [2, 3]. A non-homologous molecule, TGF-β mimic (*Hp*-TGM), released by the parasite *H. polygyrus*, could mimic TGF-β function and induce Foxp3 expression [4]. Studies on macrophage migration inhibitory factor (MIF) have shown that two MIF homologs, *Bm*MIF-1 and *Bm*-MIF-2, derived from *Brugia malayi*, have parallel functions of human MIF and regulate the host anti-inflammatory response [5].

Cytokine IL4 is a regulator of the adaptive immune response, which plays a critical role in the type 2 immune response against *Haemonchus contortus* infection [6]. Early works have reported that lambs infected with *H. contortus* could elicit an unequivocal Th2 response, exemplified by the upregulation of IL4 mRNA transcription that could be observed in the abomasum [7]. Furthermore, the significant reduction in fecal egg counts (FECs) was associated with the secretion of IL4 in the serum of infected sheep [8].

In the infection, parasitic nematodes could regulate the host immune system by excreting ESPs. In previous studies, *H. contortus* excretion and secretion products (*Hc*ESPs) inhibited the functions of goat PBMCs in vitro, particularly these molecules significantly inhibited PBMC proliferation. Strikingly, *Hc*ESPs at different stages of the life-cycle of *H. contortus* could bind to goat peripheral blood mononuclear cells (PBMCs) in vivo [9, 10]. Furthermore, recombinant *H. contortus* 14-3-3 isoform 2 (rHcftt-2) protein from *Hc*ESPs could decrease the production of IL4 [11].

Although studies have shown that *Hc*ESPs have an immunomodulatory effect on host immune responses, the functions of several individual ESP components are not yet clear. In particular, whether certain proteins or proteins from *Hc*ESPs could antagonize the function of the goat IL4 is unclear. In this study, we reported that a novel 136-aa protein, transthyretin domain containing protein (*Hc*TTR), could bind to goat recombinant IL4 (rIL4) and suppress IL4-induced PBMC proliferation and downregulate the transcription of genes in the IL4-activated JAK/STAT pathway, representing a potential antagonist against goat IL4.

## Materials and methods

### Animals and cells

Local crossbred goats without helminth infection are used for experimental animals, aged 9–12 months, fed daily with sterile feed and water, maintained in individually ventilated cages, and housed at Nanjing Agricultural University Experimental Animal Center.

Sprague Dawley (SD) rats (body weight ~ 220 g) were purchased from the Experimental Animal Center of Jiangsu, PR China (Qualified Certificate: SCXK 2008-0004).

Peripheral blood mononuclear cells were acquired from the goat jugular vein using a standard Ficoll-Hypaque (GE Healthcare, USA) gradient centrifugation method and cultured as previously described [12, 13]. Three biological replicates (three goats), each replicate consisting of three technical replicates (three replicates for each goat) were performed for cell proliferation assays and transcriptional analysis.

### Collection of HcESPs and preparation of rIL4

**Figure 1:**
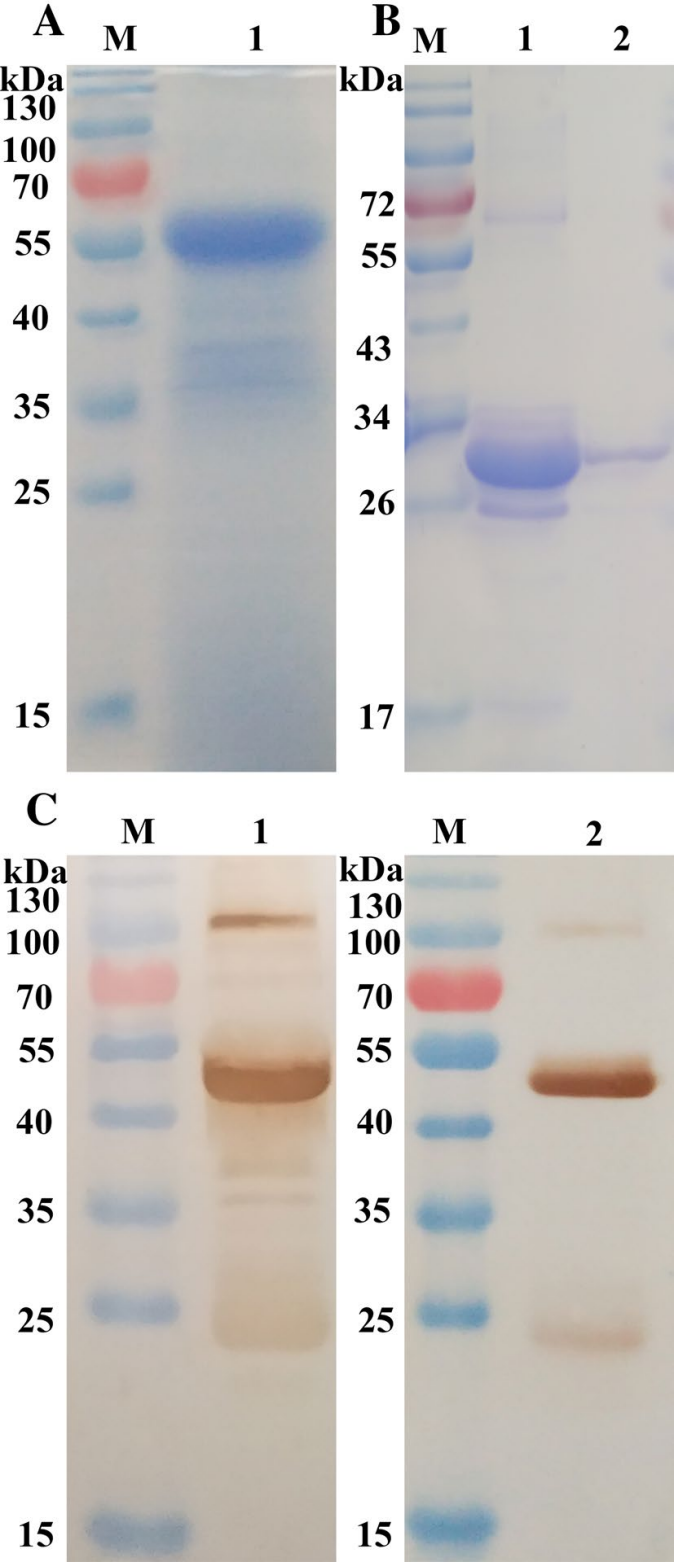
**Acquisition of** ***Hc*ESPs, rIL4 and the immunoprecipitates. A** The collection of *Hc*ESPs. M: standard protein molecular marker; Lane 1: *Hc*ESPs were resolved by SDS-PAGE on 12% polyacrylamide gel and stained with Coomassie brilliant blue R250. **B** The purification of rIL4. Protein samples were resolved by SDS-PAGE on 12% polyacrylamide gel and stained with Coomassie brilliant blue R250. M: standard protein molecular marker; Lane 1: soluble extract of cultured cells for rIL4; Lane 2: purified recombinant IL4 was approximately 30.7 kDa. **C** Western blot analysis of the interaction between *Hc*ESPs and rIL4 in vitro. Lane M: protein marker (ordinate values in kDa); Lane 1: the immunoprecipitates (rIL4 and *Hc*ESPs) obtained by Co-IP with IgG *Hc*ESPs; Lane 2: rat normal IgG was used as a control sample. The results demonstrated that *Hc*ESPs could bind to rIL4 in vitro.

*Hc*ESPs were prepared as previously described using a standard procedure [14]. Adults of *H. contortus* were collected from the abomasum of sacrificed goats and maintained in RPMI 1640 medium overnight. Subsequently, the supernatants were harvested, filtered with a 0.22 μm filter and concentrated with a 3-kDa filter (Merck Millipore, Germany). The recombinant protein and prokaryotic expression vector for goat IL4 (GenBank Acc. No. U34273) was constructed and kept in our laboratory (College of Veterinary Medicine, Nanjing Agricultural University). rIL4 promoted the proliferation of PBMCs at 25 μg/mL as described previously [15, 16]. The *Hc*ESPs and rIL4 were checked by 12% SDS-PAGE, followed by Coomassie blue staining (Figure 1). The concentrations of *Hc*ESPs and rIL4 were tested by the Bradford method [17].

### Preparations of polyclonal antibodies of HcESPs and rIL4 of goat

Polyclonal antibodies against *Hc*ESPs (IgG-*Hc*ESPs) and rIL4 (IgG-rIL4) were obtained from SD rats as previously described [18]. First, SD rats were injected with 400 μg of *Hc*ESPs or rIL4 mixed with Freund’s complete adjuvant. After 2 weeks, SD rats were immunized with Freund’s incomplete adjuvant mixed with the corresponding proteins at the same doses for four times at 1-week intervals. The sera containing specific antibodies were collected at 10 days after the last injection.

### Coimmunoprecipitation (Co-IP) and protein immunoblot assays

For the Co-IP assay, Protein A/G PLUS-Agarose Immunoprecipitation Reagent was utilized in this study (Santa Cruz Biotechnology, USA). To obtain the binding proteins of goat IL4 from *Hc*ESPs, 200 μg rIL4 and 600 μg *Hc*ESPs were incubated together at 4 °C overnight, then 2 μg rat normal IgG (Santa Cruz Biotechnology, Dallas, Texas, USA) and 40 μL Protein A/G PLUS-Agarose beads were added and incubated at 4 °C for 30 min. The beads were pelleted by centrifugation at 1000 × *g* for 5 min at 4 °C, then the precipitate was discarded, and the supernatant was divided into two equal portions: Group A and Group B. Group A was incubated with IgG-rIL4, and Group B was incubated with rat normal IgG overnight at 4 °C. Immune complexes in each group were isolated using 20 μL of protein A/G plus agarose. Immunoprecipitates were collected by centrifugation at 2500 rpm for 5 min at 4 °C. The supernatants were carefully aspirated and discarded, and the pellet was washed 4 times with phosphate-buffered saline (PBS). After the final wash, the pellets were resuspended in 1× SDS loading buffer. The immunoprecipitates obtained from Group A and Group B were used to confirm the interaction between *Hc*ESPs and rIL4 in vitro by Western blotting using IgG-*Hc*ESPs as the primary antibody.

### Liquid chromatography–tandem mass spectrometry (LC/MS–MS) analysis

The immunoprecipitates obtained from Co-IP were sent to Shanghai Applied Protein Technology, Co. Ltd. for in-solution trypsin digestion and LC/MS–MS analysis using Q Exactive (Thermo Finnigan, CA, USA). The raw files of the MS test were searched for the corresponding database using Mascot 2.2 software (v.2.2, Matrix Science, London, UK), and finally, the results of the identified proteins were obtained. The bound proteins with unique peptides greater than or equal to 2 were selected from the identified proteins to increase the confidence of the results.

### Split ubiquitin protein–protein interaction assays

The interactions between binding proteins and IL4 were further validated using a DUAL membrane pairwise interaction kit (Dual systems Biotech, Schlieren, Switzerland). Goat IL4 cloned from full-length cDNA (goat PBMCs) were inserted into bait vector pDHB1 (*LEU2*, *KanR*) (Table 1) with the C-terminal half of ubiquitin (Cub) domain. The genes of binding proteins cloned from full-length cDNA (*H. contortus*) were inserted into the prey vector pPR3N (*TRP1*, *AmpR*) with the N-terminal half of ubiquitin (Nub-G) domain. The primer sequences are given in Table 1. In addition, the gene of goat IL4 was also cloned into the vector pPR3N, and the genes of binding proteins were inserted into the vector pDHB1 for reverse validation.

**Table 1:** **Primer sequences for yeast two-hybrid screening assays**

Gene name	Primer sequence (5′-3′)
IL4	CTggccattacggccATGCACAAGTGTGACATT
	CTggccgaggcggccAAACACTTTGAGTATTTCTCCT
*Hc*ENO	ATggccattacggccATGCCTATCACGAAAA
	CTggccgcctcggccGAACTGGATTGCGGAAG
*Hc*GST	CGggccattacggccATGGTCAACTACAAGCTG
	CTggccgcctcggccGGAACGAAGTCTGGGGGC
*Hc*GB	GCggccattacggccATGTCTCCAGAAGATGTCAAG
	GCggccgcctcggccGAACGTGAGGATGTCCGTG
*Hc*TTR	CTggccattacggccATGCGGCAACAAGCCGTT
	CTggccgcctcggccGCAGGAGATCGCGTTCCTC
*Hc*Cab	ATggccattacggccATGGCCAGCCGAACTACC
	CTggccgcctcggccGCTTGAAGGGATTGGTCTT
*Hc*GAPDH	ATggccattacggccATGGTAAAACCAAAGGTTGG
	CTggccgcctcggccGGGCCTTGCTTGCAATGTA
*Hc*PEPCK	GCggccattacggccATGACCATAGACTGCATT
	GCggccgcctcggccGCATTGCATGAATTCTCTT

For interaction assays, different pairs of bait and prey vectors were co-transformed into yeast strain NMY51 using the Yeastmaker™ Yeast Transformation System 2 kit (Clontech, USA). Transformed colonies were selected in SD-LW medium and incubated for the growth of positive transformants. Several independent positive transformants were selected and recultured in SD-LW broth at 30 °C until the OD546 of the culture reached 1.0. Fifty microliters of each diluted culture (1:10, 1:100 and 1:1000) was applied to SD-LW and SD-LHAW selection plates and incubated at 30 °C for 2–3 days for protein–protein interaction assays. The plasmid pDHB1-IL4 co-transformed with the control plasmids pOstI-NubI and pPR3-N into yeast were set as controls.

### Cloning and expression of rIL4 binding proteins

For each candidate, including glutathione *S*-transferase domain containing protein (*Hc*GST); transthyretin domain containing protein (*Hc*TTR); calponin actin-binding domain containing protein (*Hc*Cab), the genes were amplified and cloned into plasmid vector pET-32a for transfection of BL-21 (DE3) competent cells. The primer sequences for PCR amplification are shown in Table 2. Recombinant proteins were obtained using a His Bind^®^ Resin Chromatography kit (Merck, Darmstadt, Germany) as previously described, and the concentration of proteins was tested by the Bradford method [11]. The cell lysate of empty pET-32a in *E. coli* was purified using the same method. Endotoxins were removed from the recombinant proteins using Detoxi-Gel Affinity Pak Prepacked columns (Pierce, Rockford, USA). The purified proteins were stored at −70 °C until further usage.

**Table 2:** **Primer sequences for PCR amplification**

Gene name	Primer sequence (5′-3′)
*Hc*GST	CGgaattcATGGTCAACTACAAGCTG (*Eco*RI)
	ATctcgagGAACGAAGTCTGGGGGC (*Xho*I)
*Hc*TTR	CTgatatcATGCGGCAACAAGCCGTT (*Eco*RV)
	ATctcgagCAGGAGATCGCGTTCCTC (*Xho*I)
*Hc*Cab	ATgaattcATGGCCAGCCGAACTACC (*Eco*RI)
	CTctcgagCTTGAAGGGATTGGTCTT (*Xho*I)

### Cell proliferation assays

IL4 has a variety of biological activities that can stimulate B cell proliferation and differentiation, as well as T cell proliferation [19, 20]. CCK-8 (Cell Counting Kit-8, Dojindo, Japan) assays were performed to identify the effects of these binding proteins in inhibiting IL4-induced PBMC proliferation. PBMCs (1 × 10^6^ cells/mL) with no treatment served as a blank group. Cells exposed to empty pET-32a protein were set as a negative control group, and cells treated with rIL4 (25 μg/mL) were set as a positive control group. PBMCs treated with a serial concentration of candidate proteins (10 μg/mL, 20 μg/mL and 40 μg/mL) were used as a control. In addition to the above control group, PBMCs exposed to rIL4 (25 μg/mL) together with candidate proteins (serial concentration: 10 μg/mL, 20 μg/mL and 40 μg/mL) were set as the experimental group, followed by incubation at 37 °C and 5% CO_2_ for 72 h under dark conditions. Then, 10 μL of CCK-8 solution was added to each well of a 96-well plate with cells. After incubation for 2 h, the absorbance values at 450 nm (OD450) were measured using a microplate reader (Thermo Scientific, USA). The cell proliferation index was calculated by the following formula: OD450 treatment groups/OD450 control. OD450 in negative controls was set as 100%. Three independent experiments were performed in this test with three technical replicates of each group.

### Real-time PCR analysis

To confirm the effects of binding proteins on goat IL4, further investigation was carried out at the molecular level. Real-time PCR was performed to detect the transcription of IL4R, JAK2 and STAT6. Group settings were consistent with the description of cell proliferation assays. The primers IL4R, JAK2 and STAT6 for qPCR are listed in Table 3. The stability of beta-actin expression, used as an endogenous reference gene, was verified. The amplification efficiencies and correlation coefficients (r^2^) of all targets and endogenous reference genes were verified to be similar by real-time PCR (Table 3). All data were obtained from the ABI Prism 7500 software (version 2.0.6; Applied Biosystems, Foster City, California, USA). Raw cycle thresholds (*Ct*) were used for the comparative *Ct* (2^−ΔΔ*Ct*^) method to calculate the level of gene expression. Three independent experiments were performed in this experiment with three technical replicates of each group.

**Table 3:** **Primer sequences for real-time PCR**

Target	NCBI Gene ID	Sequence of nucleotide (5′-3′)	Efficiency^a^	Tm (°C)	Amplicon size (bp)	Correlation coefficients (r^2^)
IL4R	102176382	CACGTCACCCACACATCATTA AAACTCCGTCTCTTCCCATTC	1.01436	58.31	129	0.9897
JAK2	102180670	AGTGCCCGTGACTCATGAAA AGATGTCCAGTGGCGTTTGA	1.107772	59.60	101	0.9977
STAT6	102180571	GGTTCAGTGACTCAGAGATTGG GGCAGAGAATGGCTGGATATT	1.069265	58.41	98	0.9997
β-Actin	102179831	CACCACACCTTCTACAAC TCTGGGTCATCTTCTCAC	1.070216	52.74	106	0.9958

^a^Amplification efficiency (%) = (10^−1/slope^ − 1).

### Statistical analysis

Statistical analysis was performed using the GraphPad Premier 6.0 software package (GraphPad Prism, San Diego, California, USA). One-way ANOVA was used for comparisons of three or more groups. Data are represented as the mean ± the standard deviation (SD). *P* values of ≤ 0.05 were defined as statistically significant. The following symbols were used to indicate the degree of significance: *P < 0.05, **P < 0.01, ****P < 0.0001; ns: non-significant. All experiments were repeated a minimum of three independent times.

## Results

### Identification of rIL4 binding proteins from HcESPs

The immunoprecipitates of rIL4 and *Hc*ESPs were collected by Co-IP. Western blot analysis of the immunoprecipitates showed that *Hc*ESPs could bind with rIL4 in vitro. In addition to the heavy and light chains of rat normal IgG, there were many clear bands in the nitrocellulose filter (NC) membrane, whereas there was no band in the negative control (Figure 1C).

The raw file of LC/MS–MS test was searched by Mascot 2.2 software, and the resulting proteins were finally identified based on the UniProt *Haemonchus* database, matching with at least two unique peptide counts and filtering by molecular weight search (MOWSE) score ≥ 20. Seven binding proteins of goat IL4 were recognized as follows: *Hc*ENO, *Hc*GST, *Hc*GB, *Hc*TTR, *Hc*Cab, *Hc*GAPDH, and *Hc*PEPCK (Table 4).

**Table 4:** **Information on the binding proteins**

Database ID	Species	Identified protein name	Unique peptides
gi|560119141	*Haemonchus contortus*	Enolase domain containing protein	11
gi|560120552	*Haemonchus contortus*	Glutathione *S*-transferase domain containing protein	5
gi|560121257	*Haemonchus contortus*	Globin domain containing protein	4
gi|560135552	*Haemonchus contortus*	Transthyretin domain containing protein	2
gi|560132282	*Haemonchus contortus*	Calponin actin-binding domain containing protein	2
gi|375076273	*Haemonchus contortus*	Glyceraldehyde-3-phosphate dehydrogenase	2
gi|560118023	*Haemonchus contortus*	Phosphoenolpyruvate carboxykinase domain containing protein	2

### Yeast two-hybrid (YTH) screening assays further validate the binding proteins of goat IL4

To further confirm the results of Co-IP, YTH screening assays were performed independently between different pairs of bait and prey vectors. The gene of goat IL4 was cloned into the C-terminal half of ubiquitin (pDHB1), and the *Hc*ENO, *Hc*GST, *Hc*GB, *Hc*TTR, *Hc*Cab, *Hc*GAPDH, and *Hc*PEPCK were successfully inserted into the N-terminal half of ubiquitin (pPR3-N).

**Figure 2:**
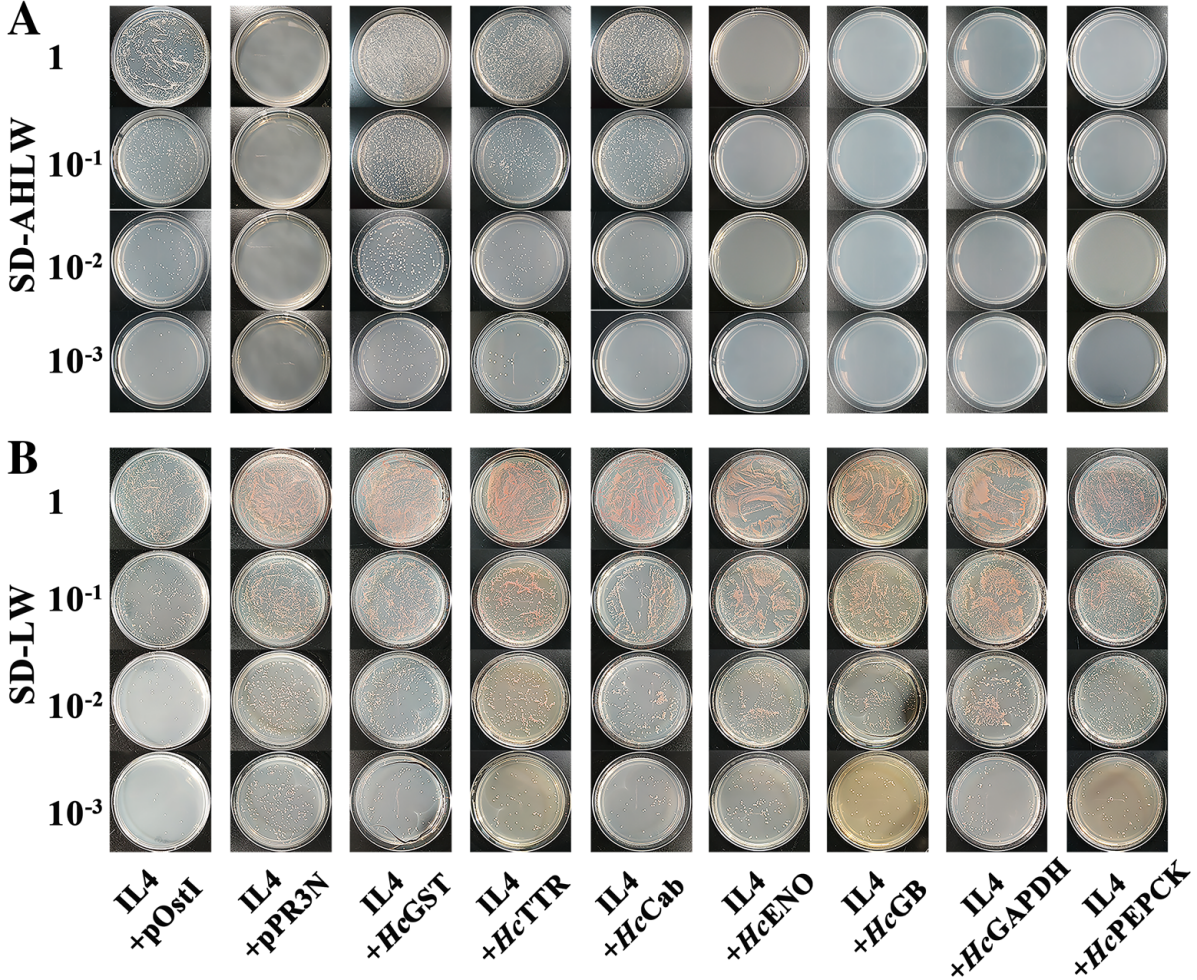
**Testing the interactions between goat IL4 and** ***Hc*ENO,** ***Hc*GST,** ***Hc*GB,** ***Hc*TTR,** ***Hc*Cab,** ***Hc*GAPDH,** ***Hc*PEPCK. A** Cells grown on selective medium SD-LW block (without Leu and Trp). **B** Cells grown on selective medium SD-AHLW block (without Ade, His, Leu and Trp). The two construct pairs of pOst I with IL4 and pPR3N with IL4 were positive control and negative control, respectively. The construct pairs of IL4 with *Hc*GST, IL4 with *Hc*TTR, IL4 with *Hc*Cab, IL4 with *Hc*PEPCK, IL4 with *Hc*ENO, IL4 with *Hc*GB, IL4 with *Hc*GAPDH were carried in NMY51. Each experiment was run in triplicate.

After co-transformation of the bait and prey vectors into NMY51, if the proteins they carried could interact with each other, which would result in the reconstruction of the split ubiquitin, then the reporter genes (HIS3 and ADE2) would allow the yeast strain to grow on SD-AHLW selective medium. When pPR3N-GST, pPR3N-TTR and pPR3N-Cab were co-transformed with pDHB1-IL4, the yeast strain NMY51 grew on SD-AHLW (Figure 2A). The results of forward tests showed that *Hc*GST, *Hc*TTR, and *Hc*Cab could actually bind with IL4, and the results of the reverse tests further illustrated the interaction of IL4 with these three proteins (Additional file 1).

### Cloning, expression and purification of HcGST, HcTTR and HcCab

**Figure 3:**
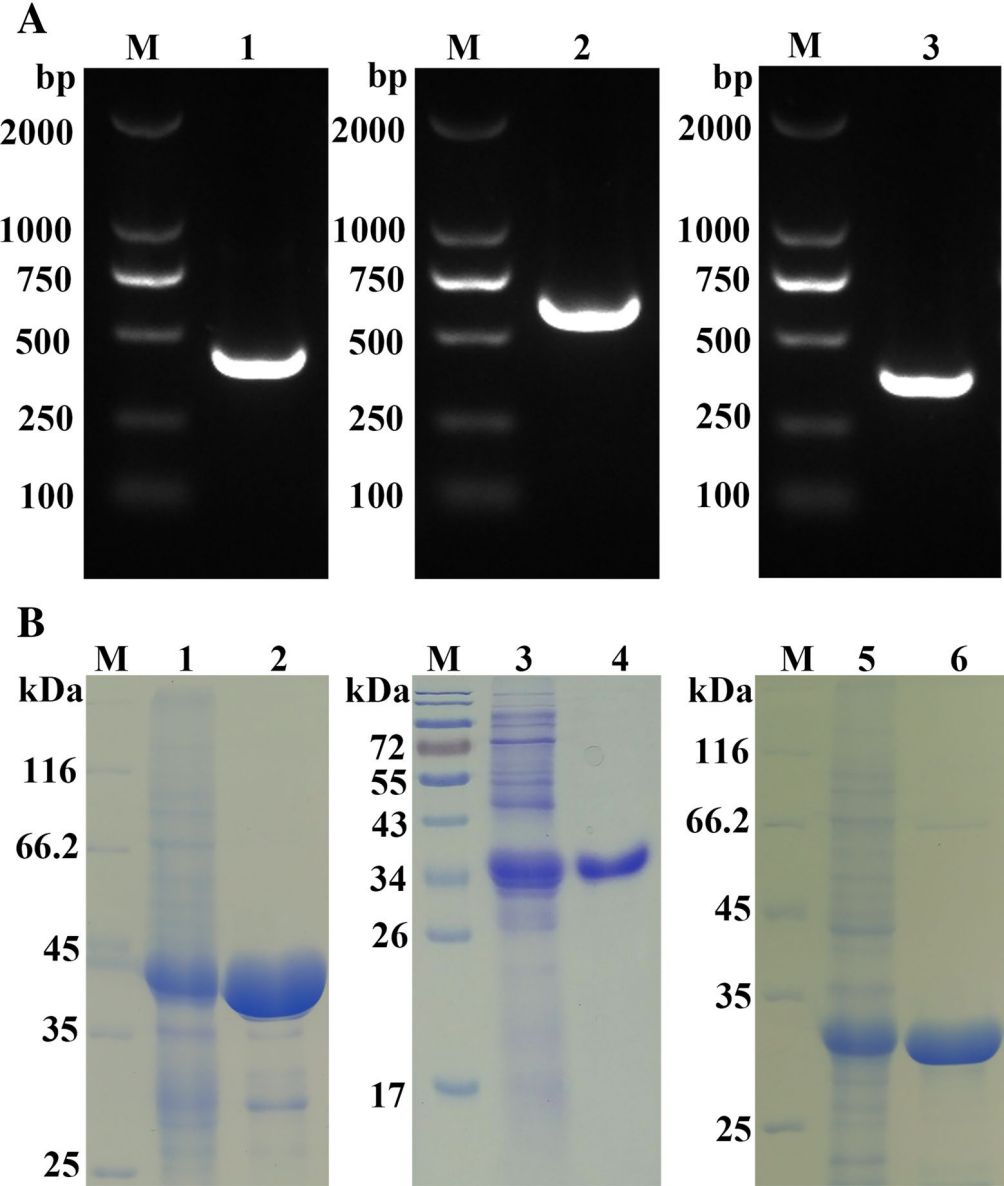
**Cloning, expression and purification of** ***Hc*GST** ***Hc*TTR and** ***Hc*Cab. A** The amplification of *Hc*GST, *Hc*TTR and *Hc*Cab. 0.1% agarose gel electrophoresis was used to detect amplification of PCR products. M: DNA standard molecular weight DL2000; Lane 1: the amplification of *Hc*GST; Lane 2: the amplification of *Hc*TTR; Lane 3: the amplification of *Hc*Cab; **B** The purification of r*Hc*GST, *rHc*TTR and *rHc*Cab. Protein samples were resolved by SDS-PAGE on 12% polyacrylamide gel and stained with Coomassie brilliant blue R250. M: Standard protein molecular marker; Lane 1: soluble extract of cultured cells for r*Hc*GST; Lane 2: purified recombinant *Hc*GST was approximately 41.2 kDa; Lane 3: soluble extract of cultured cells for r*Hc*TTR; Lane 4: purified recombinant *Hc*TTR was approximately 35.4 kDa; Lane 5: soluble extract of cultured cells for r*Hc*Cab; Lane 6: purified recombinant r*Hc*Cab was approximately 34.1 kDa.

Through YTH screening assays, three positive candidates, *Hc*GST, *Hc*TTR and *Hc*Cab, were obtained. The PCR products of the *Hc*GST, *Hc*TTR and *Hc*Cab genes were successfully amplified from the full-length cDNA of *H. contortus* (Figure 3A) and cloned into the pET-32a vector. Recombinant products of *Hc*GST (r*Hc*GST), *Hc*TTR (r*Hc*TTR) and *Hc*Cab (r*Hc*Cab) were expressed and purified by nickel chelating chromatography through affinity for the hexa-histidine tag. They were approximately 41.2 kDa, 35.4 kDa and 34.1 kDa, respectively (Figure 3B).

### rHcTTR **i**nhibited the biological function of rIL4 in vitro

**Figure 4:**
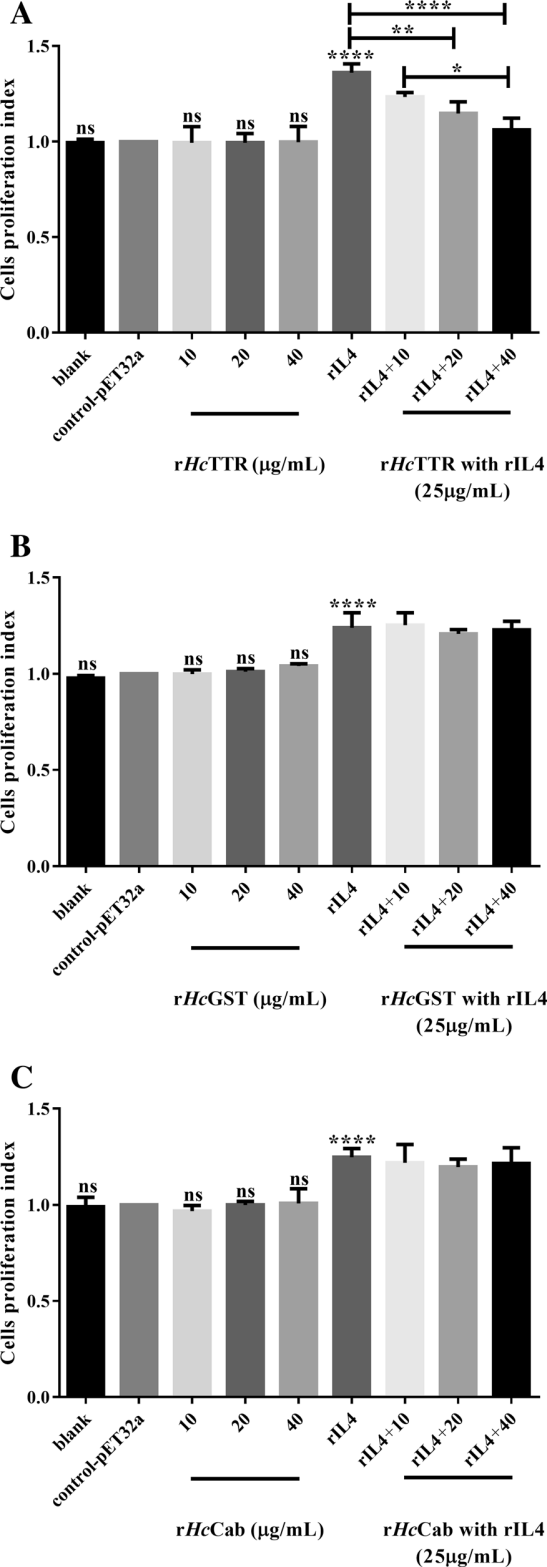
**Effects of r*Hc*GST, r*Hc*TTR and r*Hc*Cab on the biological function of rIL4. A** Cell proliferation of PBMCs was detected using CCK-8 by r*Hc*TTR co-incubated with rIL4 in vitro. **B** PBMC proliferation was detected using CCK-8 by r*Hc*GST co-incubated with rIL4 in vitro. **C** PBMC proliferation was detected using CCK-8 by r*Hc*Cab co-incubated with rIL4 in vitro. Data are presented as the mean ± SD from three independent experiments, with three technical replicates per group, ****P < 0.0001 and ns: non-significant vs the negative control group (the control-pET32a treated group) and a capped line designates two groups that differ significantly (*P < 0.05, **P < 0.01, ***P < 0.0001).

Cell proliferation assays were used to evaluate the effect of candidate molecules (r*Hc*GST, r*Hc*TTR and r*Hc*Cab) on the biological functions of rIL4. The results demonstrated that r*Hc*TTR (co-incubation with rIL4) treatments significantly suppressed the proliferation of PBMCs in a dose-dependent manner compared with the effects observed for the positive control group (ANOVA, *F* (8, 18) = 17.8, *P* < 0.0001) (Figure 4A). The other two candidate molecules had no effects (r*Hc*GST: ANOVA, *F* (8, 18) = 29.2, *P* < 0.0001) (r*Hc*Cab: ANOVA, *F* (8, 18) = 13.61, *P* < 0.0001) (Figures 4B and C).

### The interaction between rHcTTR and rIL4 affected the transcription of the JAK/STAT signaling pathway

**Figure 5:**
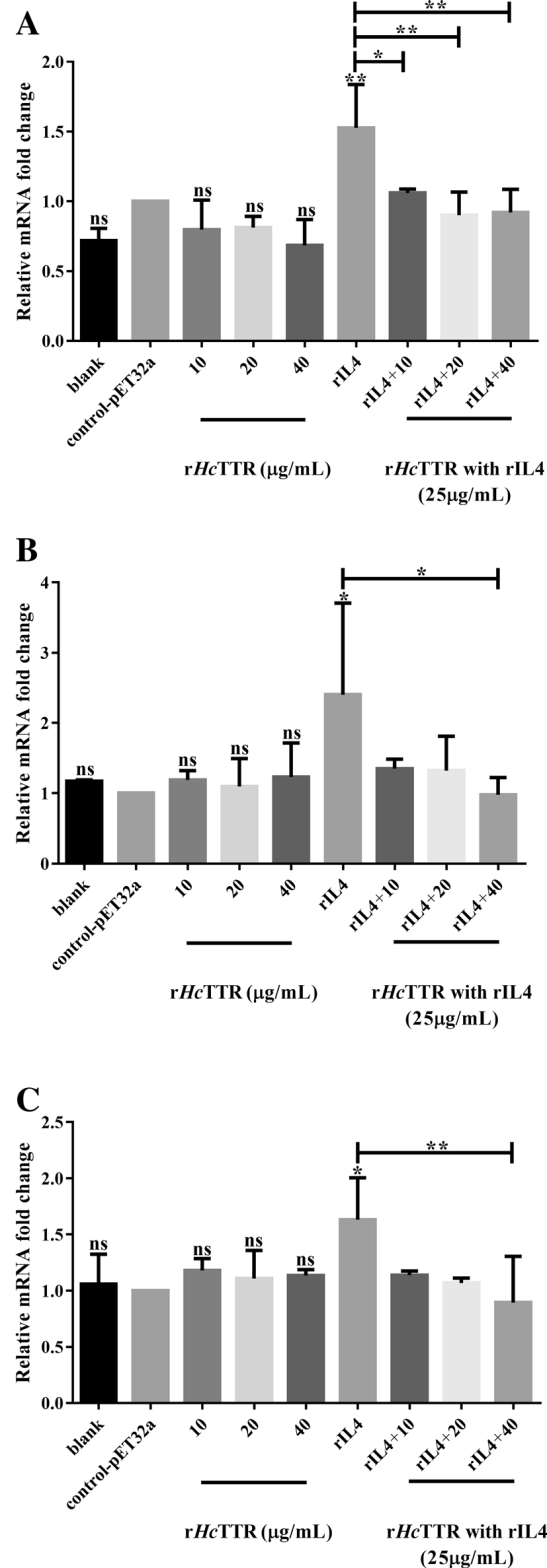
**Co-incubation of r*Hc*TTR and rIL4 affected the mRNA expression of IL4R, JAK2 and STAT6 in PBMCs. A** Transcription analysis of IL4R in goat PBMCs. **B** Transcription analysis of JAK2 in goat PBMCs. **C** Transcription analysis of STAT6 in goat PBMCs (*P < 0.05; **P < 0.01). Data are presented as the mean ± SD from three independent experiments, with three technical replicates per group, ****P < 0.0001 and ns: non-significant vs the negative control group (the control-pET32a treated group), and a capped line designates two groups that differ significantly (*P < 0.05, **P < 0.01, ****P < 0.0001).

Effects of r*Hc*TTR co-incubated with rIL4 on the gene expression of IL4R, JAK2 and STAT6 were analyzed by real-time PCR. As indicated in Figure 5, r*Hc*TTR co-incubated with rIL4 significantly decreased the transcription of IL4R (ANOVA, *F* (8, 18) = 7.057 *P* = 0.0003) (Figure 5A). The transcription of JAK2 was apparently suppressed (ANOVA, *F* (8, 18) = 2.070, *P* = 0.0954) by r*Hc*TTR co-incubated with rIL4 (Figure 5B). The transcription of STAT6 was prominently suppressed (ANOVA, *F* (8, 18) = 2.479, *P* = 0.0523) by r*Hc*TTR co-incubated with rIL4 (Figure 5C).

## Discussion

IL4 is regarded as a prominent feature of the Th2-dominated immune response and plays an important role in preventing parasitic nematode infection. Many studies have suggested that nematode infection can induce a Th2 response and result in strong IL4 production [21]. Studies have demonstrated that antibodies against IL4 or anti-IL4 receptors blocked protective immunity against parasites [22]. Therefore, the identification of IL4 antagonist molecules was of great significance.

Previous studies on *Hc*ESPs demonstrated that either *Hc*ESPs or a single component of *Hc*ESPs could affect host immune functions [10, 14, 23, 24], such as rMiro-1, rHc-AK, rFg14-3-3e, and rHCcyst-3 could regulate the function of goat PBMCs in vitro [25–28]. Moreover, the recombinant MIF from *H. contortus* (r*Hc*MIF-1) could adjust multiple functions of goat monocytes [29]. In the current study, we identified that a variety of *Hc*ESP proteins could bind to cytokine IL4 by co-IP assays. This result indicated that the antagonists of goat IL4 probably existed in *Hc*ESPs.

The yeast two-hybrid assays in this research further confirmed that *Hc*GST and *Hc*TTR were binding proteins to IL4. Previous studies have shown that GSTs from *Trichinella spiralis*, *Schistosoma mansoni*, and bovine filarial parasite (*Setaria cervi*) were immunogenic and could elicit protective immunity [30–32]. *Hc*TTR, with partial similarity to transthyretin, contained a TTR-52 domain belonging to the transthyretin-like (TTL) family. TTLs have been confirmed to be widespread in nematode genes (e.g., *H. contortus*, *Caenorhabditis elegans*, *Ostertagia ostertagi*, *B. malayi* and *Ancylostoma caninum*) [14, 33–36]. Previous studies have demonstrated that the TTLs from ESP products of *H. contortus* could be recognized by the serum from naturally immunized sheep [14] and had vaccine potential [36]. These results indicated that *Hc*GST and *Hc*TTR played important roles in the immunity and immune regulation of nematode infections. *Hc*Cab belongs to the Calponin superfamily but its role in immunity is still unclear and needs to be further researched.

Cell proliferation assays showed that rIL4 could promote the proliferation of goat PBMCs. r*Hc*TTR alone could not increase or decrease the proliferation of the cells compared with the control. However, co-incubation of r*Hc*TTR with rIL4 significantly inhibited the proliferation of PBMCs in a dose-dependent manner. Conversely, r*Hc*GST and r*Hc*Cab did not decrease the promotion of IL4 to cell proliferation. This result indicated that the binding of r*Hc*TTR to IL4 could block the function of IL4 to induce cell proliferation.

The JAK–STAT signaling pathway is a signal transduction pathway stimulated by cytokines, which is involved in many important biological processes, such as cell proliferation, differentiation, apoptosis and immune regulation [37–39]. IL4 can provoke the JAK–STAT signaling pathway associated with the IL4 receptor [40]. In this study, transcription analysis revealed that the gene transcription of IL4R, JAK2 and STAT6 was significantly downregulated in the co-incubation group of r*Hc*TTR and rIL4 compared with the other groups. These results indicated that r*Hc*TTR could impede the activation of this pathway by IL4. This result, together with the yeast two-hybrid assay and the cell proliferation assay, suggested that r*Hc*TTR could significantly decrease the functions of IL4 to induce cell proliferation in vitro and was an antagonist of goat IL4.

In our study, we confirmed that the *Hc*TTR of *Hc*ESPs was an antagonist of goat IL4. The recombinant protein could bind to goat rIL4 and significantly inhibit the biological activity of rIL4 on goat PBMC proliferation and block the activation of the JAK–STAT signaling pathway by IL4 in vitro. This study might reveal a new mechanism for parasite immune evasion. However, the functions of the other binding proteins to IL4 identified by Co-IP and yeast two-hybrid assays should be further investigated.

## Additional file

**Additional file 1.** **Reverse test for the interaction between goat IL4 and** ***Hc*ENO,** ***Hc*GST,** ***Hc*GB,** ***Hc*TTR,** ***Hc*Cab,** ***Hc*GAPDH,** ***Hc*PEPCK. A** Interaction between *Hc*ENO and IL4. **B** Interaction between *Hc*GST and IL4. **C** Interaction between *Hc*GB and IL4. **D** Interaction between *Hc*TTR and IL4. **E** Interaction between *Hc*Cab and IL4. **F** Interaction between *Hc*GAPDH and IL4. **G** Interaction between *Hc*PEPCK and IL4. The vectors pOstI and pPR3N were set as positive controls and negative controls, respectively. The construct pairs of *Hc*ENO with IL4, *Hc*GST with IL4, *Hc*GB with IL4, *Hc*TTR with IL4, *Hc*Cab with IL4, *Hc*GAPDH with IL4, and *Hc*PEPCK with IL4 were carried in NMY51. Each experiment was run in triplicate.

## Abbreviations

Co-IP: co-immunoprecipitation
*Hc*ESPs: *Haemonchus contortus* excretory and secretory products
IL4: interleukin 4
LC/MS–MS: liquid chromatography–mass spectrometry/mass spectrometry
MIF: macrophage migration inhibitory factor
PBMCs: peripheral blood mononuclear cells
SD: Sprague–Dawley
Th2: T-helper cell type 2
YTH: yeast two-hybrid
