# Supplementary figures and images for: HcTTR: a novel antagonist against goat interleukin 4 derived from the excretory and secretory products of Haemonchus contortus
Source: Vet Res. 2019 Jun 4;50:42. doi: 10.1186/s13567-019-0661-z (PMC6549353; doi:10.1186/s13567-019-0661-z)

**
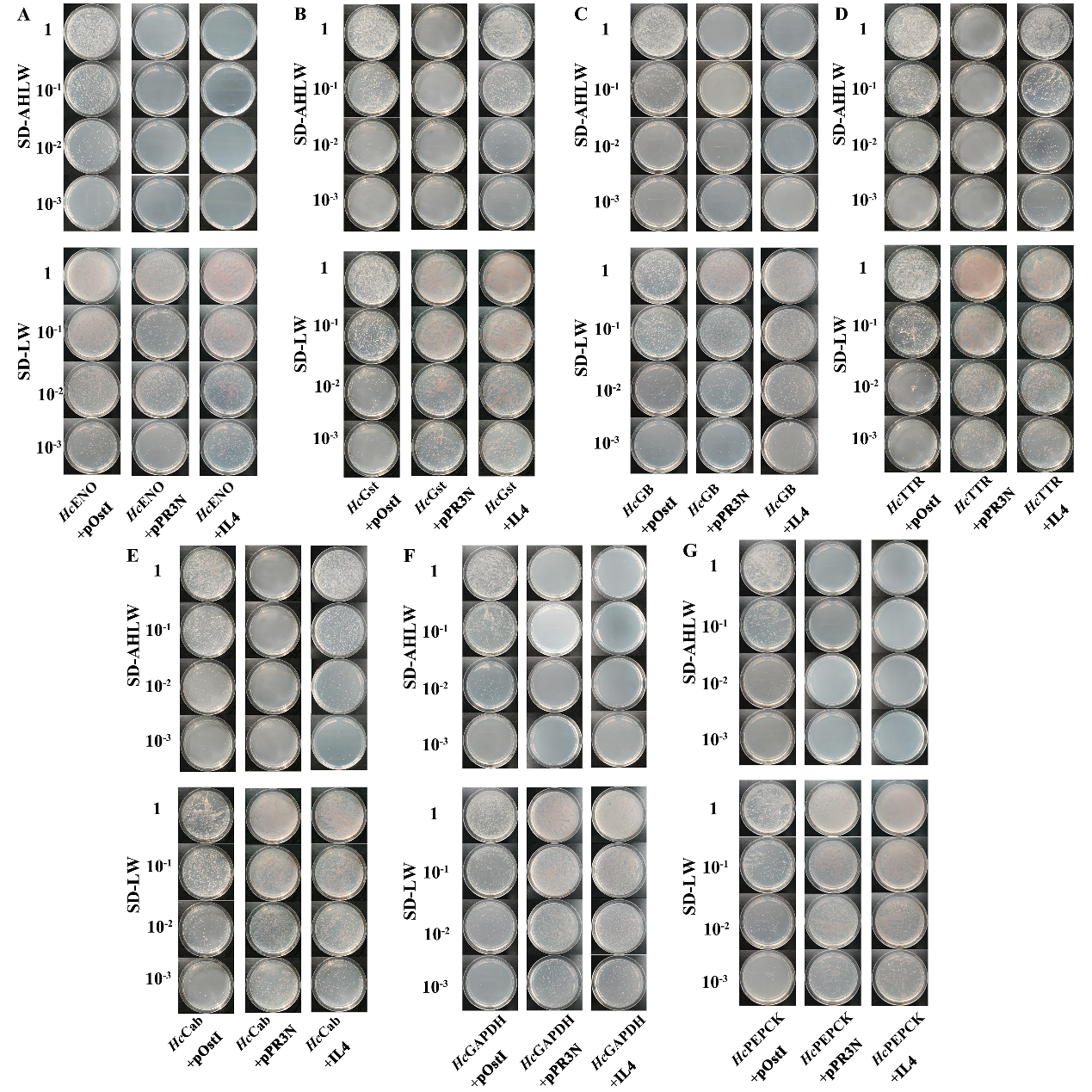
**

Supplement: Supplementary file 1 — Additional file 1. Reverse test for the interaction between goat IL4 and HcENO, HcGST, HcGB, HcTTR, HcCab, HcGAPDH, HcPEPCK. A Interaction between HcENO and IL4. B Interaction between HcGST and IL4. C Interaction between HcGB and IL4. D Interaction between HcTTR and IL4. E Interaction between HcCab and IL4. F Interaction between HcGAPDH and IL4. G Interaction between HcPEPCK and IL4. The vectors pOstI and pPR3N were set as positive controls and negative controls, respectively. The construct pairs of HcENO with IL4, HcGST with IL4, HcGB with IL4, HcTTR with IL4, HcCab with IL4, HcGAPDH with IL4, and HcPEPCK with IL4 were carried in NMY51. Each experiment was run in triplicate. [file 13567_2019_661_MOESM1_ESM.docx]
